# Phylogenomic Evidence for the Origin of Obligate Anaerobic Anammox Bacteria Around the Great Oxidation Event

**DOI:** 10.1093/molbev/msac170

**Published:** 2022-08-03

**Authors:** Tianhua Liao, Sishuo Wang, Eva E Stüeken, Haiwei Luo

**Affiliations:** Simon F. S. Li Marine Science Laboratory, School of Life Sciences and State Key Laboratory of Agrobiotechnology, The Chinese University of Hong Kong, Shatin, Hong Kong SAR 999077, China; Simon F. S. Li Marine Science Laboratory, School of Life Sciences and State Key Laboratory of Agrobiotechnology, The Chinese University of Hong Kong, Shatin, Hong Kong SAR 999077, China; School of Earth and Environmental Sciences and Centre for Exoplanet Science, University of St Andrews, Bute Building, Queen’s Terrace KY16 9TS, United Kingdom; Simon F. S. Li Marine Science Laboratory, School of Life Sciences and State Key Laboratory of Agrobiotechnology, The Chinese University of Hong Kong, Shatin, Hong Kong SAR 999077, China

**Keywords:** anammox bacteria, molecular dating analysis, planctomycetes

## Abstract

The anaerobic ammonium oxidation (anammox) bacteria can transform ammonium and nitrite to dinitrogen gas, and this obligate anaerobic process accounts for up to half of the global nitrogen loss in surface environments. Yet its origin and evolution, which may give important insights into the biogeochemistry of early Earth, remain enigmatic. Here, we performed a comprehensive phylogenomic and molecular clock analysis of anammox bacteria within the phylum Planctomycetes. After accommodating the uncertainties and factors influencing time estimates, which include implementing both a traditional cyanobacteria-based and a recently developed mitochondria-based molecular dating approach, we estimated a consistent origin of anammox bacteria at early Proterozoic and most likely around the so-called Great Oxidation Event (GOE; 2.32–2.5 Ga) which fundamentally changed global biogeochemical cycles. We further showed that during the origin of anammox bacteria, genes involved in oxidative stress adaptation, bioenergetics, and anammox granules formation were recruited, which might have contributed to their survival on an increasingly oxic Earth. Our findings suggest the rising levels of atmospheric oxygen, which made nitrite increasingly available, was a potential driving force for the emergence of anammox bacteria. This is one of the first studies that link the GOE to the evolution of obligate anaerobic bacteria.

## Introduction

Anaerobic ammonium oxidation (anammox, NH_4_^+^ + NO_2_^–^ → N_2_ + 2H_2_O) (Broda 1977), which usually occurs in anoxic marine, freshwater, and wetland settings, accounts for up to 50% of the removal of fixed nitrogen (N) in nature. Along with denitrification, it is recognized as an important biological process that leads to N loss from the environment (Oshiki et al. 2016; Stein and Klotz 2016). In wastewater treatment, anammox is more cost-effective and environmentally friendlier than denitrification due to its lower oxygen requirement for aeration [nitrite (N{+III}), instead of nitrate (N{+V}) is sufficient for the anammox metabolism], carbon-free cultivation (most denitrifying bacteria are heterotrophic), and its negligible emissions of greenhouse gases like N_2_O (Jetten et al. 2001; Van Dongen et al. 2001). Consequently, anammox bacteria have been widely used in wastewater treatment plants (Gao and Tao 2011; Okabe et al. 2011; Stein and Klotz 2016; Li, Ling et al. 2020). Despite the environmental and industrial importance of these organisms, their evolutionary history and antiquity are poorly known, which hinders accurate reconstructions of the biogeochemical nitrogen cycle over geologic time.

Previous studies investigated the roles of key genes driving anammox, including *hzsCBA* (hydrazine synthase) (Dietl et al. 2015), *hdh* (hydrazine dehydrogenase), *hao* (hydroxylamine oxidoreductase) (Kartal, Maalcke et al. 2011), and *nxr* (nitrite oxidoreductase) (Chicano et al. 2021). In general, the vital enzymes encoded by these genes either are directly involved in anammox or participate in replenishing electrons to the cyclic electron flow (Kartal and Keltjens 2016; Hu et al. 2019). Furthermore, all known anammox bacteria are found within the phylum Planctomycetes (Wang et al. 2019). Seven candidate genera of anammox bacteria, namely “*Candidatus* Brocadia”, “*Candidatus* Kuenenia”, “*Candidatus* Jettenia”, “*Candidatus* Scalindua”, “*Candidatus* Anammoximicrobium”, “*Candidatus* Anammoxoglobus” and “*Candidatus* Bathyanammoxibiaceae” have been proposed based on 16S ribosomal RNA (rRNA) gene sequences (Zhang and Okabe 2020; Zhao et al. 2022), but none of them have been successfully isolated into pure cultures. The habitat of anammox bacteria requires the simultaneous presence of reduced (ammonia) and oxidized (nitrite) inorganic N compounds. Such habitats are often found at the aerobic–anaerobic interface in aquatic ecosystems, including the margins of oxygen minimum zones (OMZs) in the ocean and sediment–water interfaces, where ammonium originates from the anaerobic degradation of organic matter and nitrite can be produced by aerobic ammonia oxidation (Sonthiphand et al. 2014). Ammonium, one of the two substrates of the anammox metabolism, is thermodynamically stable and was likely present in moderate concentrations in the deep ocean throughout the Archean (Yang et al. 2019) and probably extending well into the Proterozoic (Stueken et al. 2016). The availability of nitrite, the second important substrate, is less certain. Geochemical data from sedimentary rocks suggest that the early Earth (before 3 Ga) was fully anoxic and deficient in aerobic ecosystems that were able to generate nitrite/nitrate (Stueken et al. 2015; Koehler et al. 2019; Ossa Ossa et al. 2019). The first transient and/or localized occurrences of aerobic nitrogen cycling appear in the Mid- to Neoarchean around 2.7 Ga (Garvin et al. 2009; Godfrey and Falkowski 2009; Homann et al. 2018; Koehler et al. 2018), but evidence of widespread nitrate availability does not appear until around 2.4 Ga, the so-called Great Oxidation Event (GOE) (Zerkle et al. 2017; Kipp et al. 2018; Luo et al. 2018). The GOE marks the rise of free molecular oxygen (O_2_) in the atmosphere above 10^−5^ times modern levels, and was ultimately a result of the emergence of oxygenic cyanobacteria (Lyons et al. 2014). Hence it is conceivable that the appearance of anammox was linked to the appearance of aerobic ecosystems around the time of the GOE. However, it is also possible that some nitrite was provided much earlier, through lightning reactions in the Archean atmosphere (Navarro-González et al. 1998). Lightning, even in the absence of O_2_, can generate nitric oxide (NO) gas, which dissolves in water and converts into aqueous species, including nitrite (Kasting and Walker 1981). Some anammox bacteria are even capable of using NO rather than nitrite directly as a substrate (Hu et al. 2019). If these organisms capitalized on the lightning flux, then anammox might long pre-date the GOE.

Several tools exist for investigating the link between the evolution of metabolic pathways and geo-environmental transformations. One way to explore the evolutionary history of a specific metabolic pathway is based on organic biomarkers, which, however, are often affected by the poor preservation over geologic timescales (Hallmann et al. 2011). For instance, ladderanes, a type of lipids that is unique to anammox bacteria (Moss et al. 2018), are rarely preserved to a level that can be used to date their evolutionary origin. Another approach is based on nitrogen isotope ratios of sedimentary records (Ader et al. 2016; Mettam et al. 2019; Yang et al. 2019). However, the isotopic fractionation factors for different metabolic pathways overlap widely [denitrification: –5 to –30‰; anammox: –16 or –24‰ (Brunner et al. 2013; Stueken et al. 2016)], making it difficult to single out and elucidate the evolution of the different redox reactions within the N cycle by this method (Fuchsman and Stueken 2021). Alternatively, molecular dating, which estimates the age of the last common ancestor (LCA) of analyzed lineages by comparing their sequences based on the molecular clock theory (Yang and Rannala 2006), provides an alternative strategy to investigate this issue. Briefly, it can estimate the divergence timescale of organisms using genetic data while accounting for issues like uncertainties in the calibrations and different evolutionary rates among lineages, thereby bypassing the paucity and uncertainty of biomarkers and other biogeochemical records. On the one hand, because anammox is an anaerobic reaction, it is intuitive to assume that anammox should originate before the GOE, perhaps using nitrite or NO provided by lightning reactions in the atmosphere (Ducluzeau et al. 2009). On the other hand, it is because of GOE that O_2_ on Earth rose permanently to a concentration that is biologically meaningful, and as a result, (micro-)environments were created in the surface ocean that were rich in nitrate/nitrite produced by aerobic organisms. These redox-stratified conditions may have stimulated the anammox metabolism by providing both nitrite and ammonium at the redoxcline. In this regard, it could be hypothesized that anammox did not arise until the GOE. Here, we ask the question when anammox originated. To answer this question and to obtain more insights into the evolution of anammox bacteria, we compiled an up-to-date genomic dataset of Planctomycetes (see supplementary text section 1; supplementary dataset S1.1, Supplementary Material online), placed the evolution of anammox bacteria into the context of geological events, and investigated genomic changes associated with the origin of this ecologically important bacterial lineage.

## Results and Discussion

### Monophyletic Origins of Anammox Bacteria and Anammox Genes

The anammox bacteria form a monophyletic group within the phylum Planctomycetes in a comprehensive phylogenomic tree with 881 Planctomycetes genomes (supplementary fig. S1, Supplementary Material online). The anammox bacteria clade in this phylogenomic tree (supplementary fig. S1, Supplementary Material online) comprises four known genera including the early-branching *Ca.* Scalindua and *Ca.* Kuenenia, and the late-branching *Ca.* Jettenia and *Ca.* Brocadia. It also includes two novel lineages which we named “basal lineage” (named as *Ca.* Bathyanammoxibiaceae in a recent study [Zhao et al. 2022]) and “*hzsCBA*-less lineage” (supplementary fig. S1, Supplementary Material online), both of which have relatively low genome completeness compared to the other anammox bacteria and are represented by metagenome-assembled genomes (MAGs) sampled from groundwater (see supplementary text section 2.1, Supplementary Material online). Note that the absence of *hzsCBA* in several genomes within the “*hzsCBA*-less lineage” might be ascribed to the loss of these genes in evolution or their low genome completeness (supplementary fig. S1, Supplementary Material online). Because of their shallow (late-branching) phylogenetic position, this uncertainty should not affect the inference of the origin of anammox bacteria. Two described genera (*Ca.* Anammoximicrobium and *Ca.* Anammoxoglobus) do not have genomes available by the time of the present study (last accessed: April 2021), and are therefore not included in the phylogenomic analysis. Furthermore, using the last updated set of 2,077 Planctomycetes genomes (retrieved in April 2021) from NCBI, we obtained a consistent topology of anammox bacteria phylogeny (supplementary fig. S2, Supplementary Material online).

We further built a 16S rRNA gene tree (supplementary fig. S3, Supplementary Material online) using the identified 16S rRNA genes from genomic sequences of anammox bacteria and the deposited 16S rRNA gene amplicons in SILVA from the class Brocadiae, to which anammox bacteria belong (see supplementary text section 2.2, Supplementary Material online). Since the anammox bacteria with genome sequences in this 16S rRNA gene tree (supplementary fig. S3, Supplementary Material online) showed a branching order congruent with the topology of the phylogenomic tree (supplementary fig. S1, Supplementary Material online), and since the 16S rRNA gene tree included 913 (clustered from 20,142) sequences sampled from a wide array of habitats (marine, sediments, man-made reactor, and freshwater and terrestrial ecosystems), the 16S rRNA gene phylogeny, which could better capture the diversity of anammox bacteria by including uncultured samples, likely encompasses the early-branching lineages of anammox bacteria. reconciliation (see supplementary text section 2.3, Supplementary Material online) of the phylogenies of the genes critical to the anammox reaction (*hzsCBA*) (Kartal, Maalcke et al. 2011; Harhangi et al. 2012) and the genome-based species phylogenies pointed to a single origin of anammox metabolism at the LCA of anammox bacteria (supplementary fig. S4, Supplementary Material online). The above analyses suggest that our genome datasets have encompassed the earliest-branching lineages and deep phylogenetic diversity of anammox bacteria, and indicate its monophyletic origin, which agrees with previous studies (Schmid et al. 2003; Terada et al. 2011; Hamasaki et al. 2018). This result is not surprising, as a complex enzyme like hydrazine synthase is unlikely to have multiple origins, but it provides a foundation for estimating the origin of anammox metabolism by estimating the origin time of anammox bacteria.

### The Evolutionary Origin of Anammox Bacteria Coincided with the Rising O_2_

**Fig. 1.**
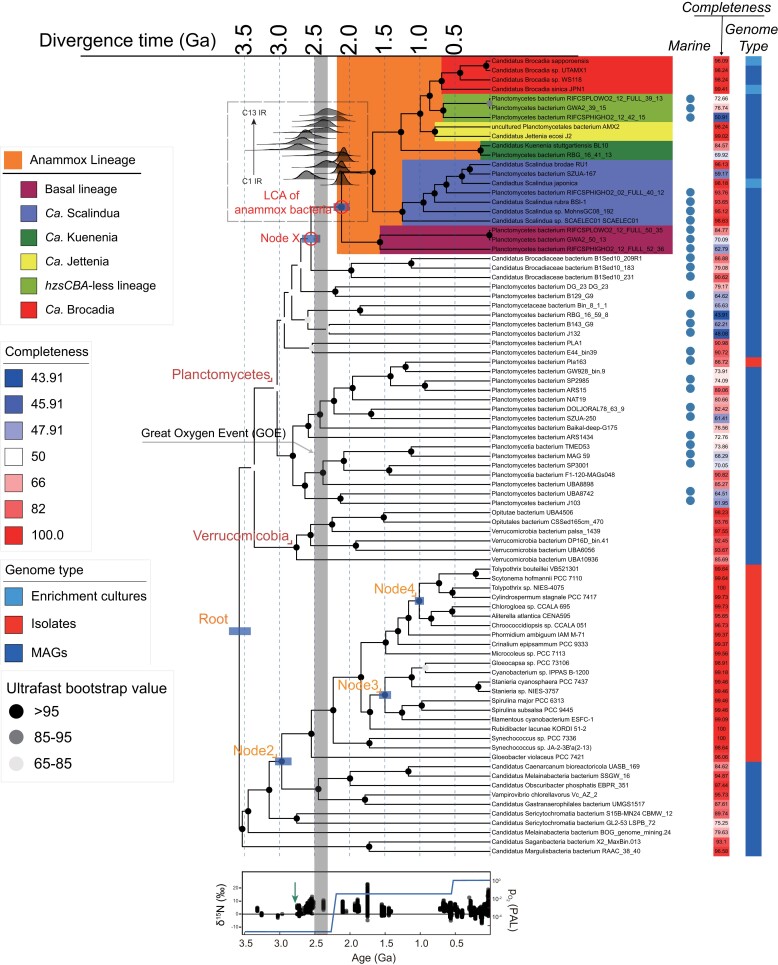
The evolutionary timeline of anammox bacteria using MCMCTree. The chronogram was estimated based on the calibration set C1 (see supplementary text section 3, Supplementary Material online) and sequence alignments of the 25 orthologs conserved in bacteria (20,040 nucleotide sites after removing the third position of codon). The blue bars on the four calibration nodes and the LCA of anammox bacteria represent the posterior 95% HPD interval of the posterior time estimates. These alternative calibration sets were selected by choosing the three that accommodate different calibration constraints (see supplementary text section 3.3, Supplementary Material online). More alternative time estimates are provided in supplementary fig. S4, Supplementary Material online. The vertical grey bar represents the period of the GOE from 2,500 to 2,320 Ma. The calibration constraints used within the phylum Cyanobacteria are marked with orange texts: the LCA of Planctomycetes and Cyanobacteria (Root), the total group of oxygenic Cyanobacteria (Node2), the total group of Nostocales (Node3), and the total group of Pleurocapsales (Node4). Planctomycetes from marine or groundwater habitats were labeled by a blue circle. The genome completeness estimated by CheckM is visualized with a gradient color strip. The right next color strip indicates the genome type of genomic sequences used in our study including MAGs and whole-genome sequencing (WGS) from either enriched culture sample or isolate. The different posterior distributions of the age of the LCA of anammox bacteria obtained using different calibrations and IR clock model (see supplementary dataset S2.1, Supplementary Material online) are indicated adjacent to the corresponding node. The diagram below the dated tree illustrates the change of atmospheric partial pressure of O_2_ (*P*O_2_) and nitrogen isotope fractionations. The blue line shows the proposed model according to Lyons et al. (2014). The green arrow suggests the earliest evidence for aerobic nitrogen cycling at around 2.7 Ga. PAL on right axis means *P*O_2_ relative to the present atmospheric level. The black dots denote the change of nitrogen δ^15^*N* isotope values according to Kipp et al. (2018).

The paucity of lineage-specific bacterial fossils makes dating deep-time microbial evolution very challenging. There are two approaches to our knowledge: the cyanobacteria-fossil-based approach, where cyanobacteria fossils were used as the only calibrations, and the mitochondria-based approach, which is based on mitochondrial endosymbiosis and can benefit from the abundant eukaryotic fossils as calibrations. We first employed a cyanobacteria-fossil-based strategy, a widely used strategy in dating deep-time bacterial evolution (Sanchez-Baracaldo et al. 2017; Wang et al. 2020). Using the dating scheme C1 as the major calibration (fig. 1; see supplementary text section 3, Supplementary Material online), we dated the origin of anammox bacteria to 2,117 Ma (95% highest posterior density [HPD] interval, 2,002–2,226 Ma). Furthermore, we repeated the analysis based on an expanded Planctomycetes genomes (Genome set 2; supplementary dataset S1.1, Supplementary Material online) and the constraint topology inferred with a profile mixture model (LG + C60 + G; Genome set 1; supplementary dataset S1.1, Supplementary Material online). In general, both analyses based on different genome sets resolved consistent topologies (supplementary fig. S5, Supplementary Material online) as shown in Figure 1. Overall, they gave similar posterior times of the LCA of the anammox bacteria at 2,105 Ma (95% HPD: 1,961–2,235 Ma) or 2,005 Ma (95% HPD: 1,869–2,146 Ma).

Analyses with different combinations of calibrations and parameters broadly converge to similar time estimates (supplementary fig. S6; supplementary dataset S2.1, Supplementary Material online). Specifically, assigning a more ancient time constraint as the minimum time bound of the total group of oxygenic cyanobacteria, which is based on the biogeochemical evidence of the presence of O_2_ at nearly 3.0 Ga (Betts et al. 2018) instead of GOE (supplementary dataset S2.1, Supplementary Material online), shifted the posterior dates of the LCA of anammox bacteria into the past by ∼22 My (e.g., C6 vs. C12; supplementary fig. S6, Supplementary Material online). Varying the maximum time bounds of the root by using 4.5, 3.8, or 3.5 Ga had a minor impact (around 100 My) on the time estimates of anammox bacteria (e.g., comparing C1, C3, and C5; supplementary fig. S6, Supplementary Material online). Note that the use of these different maximum bounds is just to test the impact of different root maxima on the posterior dates but does not necessarily mean that they are based on solid biological evidence as it is difficult to set a convincing maximum age of the root (Marshall 2019), that is, the LCA of Planctomycetes and Cyanobacteria (see supplemental text section 3.2, Supplementary Material online). Another important source of uncertainty in date estimates is the choice of the clock model (Dos Reis et al. 2016). Though the auto-correlated rate (AR) model was less favored than the independent rate (IR) model based on the model comparison (see supplementary text section 3.3; supplementary dataset S2.2, Supplementary Material online), it is worth noting that the posterior ages estimated by the AR model were 1–12% younger than estimated by the IR model (supplementary dataset S2.1, Supplementary Material online).

**Fig. 2.**
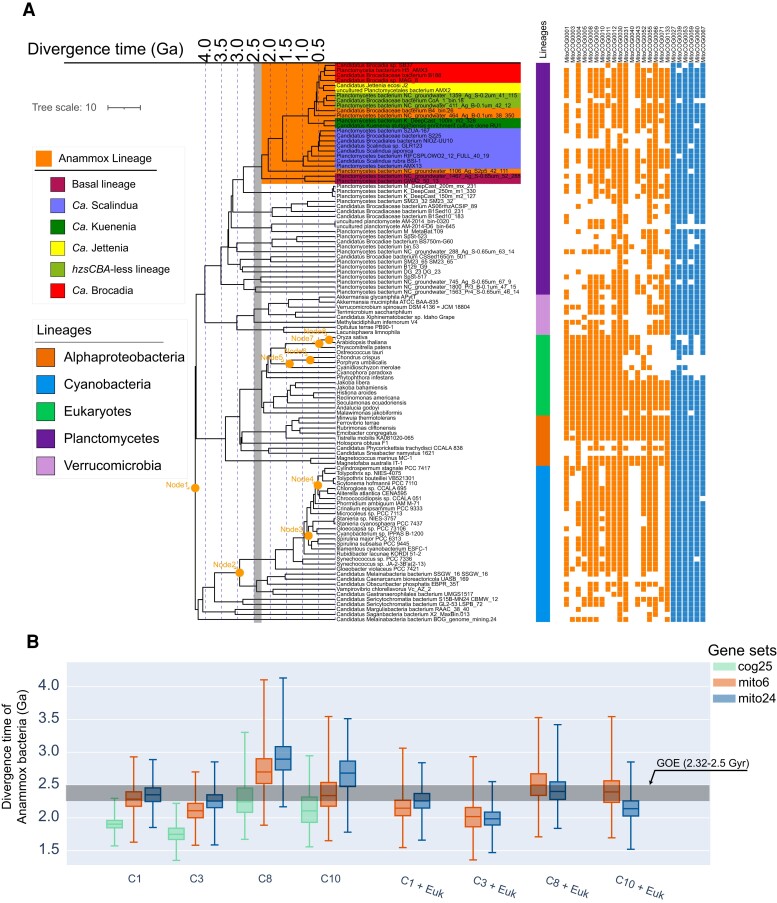
(*A*) The chronogram of the Anammox lineage estimated by mitochondria-based dating analysis. The time tree was estimated based on the calibration set “C1 + Euk” and sequence alignments of the 24 mitochondria-encoded proteins at the amino acid level (6,295 amino acid sites). The vertical grey bar represents the period of the GOE from 2,500 to 2,320 Ma. The calibration constraints are marked with orange texts: the LCA of Planctomycetes and Cyanobacteria (Root), the total group of oxygenic Cyanobacteria (Node2), the total group of Nostocales (Node3), the total group of Pleurocapsales (Node4), the total group of Bangiales (crown group of red algae) (Node5), the total group of Florideophyceae (Node6), the total group of mosses (crown group of Embryophyta) (Node7), and the total group of eudicots (crown group of angiosperms) (Node8). The color strip next to the label represent the major lineages of analyzed genomes. The filled and empty squares, respectively, represent the presence and absence of particular genes used in molecular dating analysis. The six genes selected to comprise the mito6 gene set are represented by blue squares. (*B*) The posterior times of anammox bacteria estimated using cyanobacteria-based and mitochondria-based dating analyses. The calibration sets starting with C represent the calibration sets with cyanobacterial calibrations, while those starting with Euk represent the calibration sets with eukaryotic calibrations. Calibration information: calibration scheme C1 Node1: <4.5 Ga, Node2: 3.0–2.32 Ga; C3 Node1: <3.8 Ga, Node2: 3.0–2.32 Ga; C8 Node1: <4.5 Ga, Node2: >3.0 Ga; C10 Node1: <3.8 Ga, Node2: >3.0 Ga. All of these calibration schemes share the same calibration for Nodes 3 (>1.6 Ga) and 4 (>1.7 Ga). The horizontal grey bar represents GOE from 2.5 to 2.32 Ga. The detailed constraints of calibrations and time estimates are provided in supplementary dataset S2.1, Supplementary Material online.

While cyanobacteria fossils are the most widely used calibrations (and in many times the only calibrations) in bacterial molecular dating studies (Battistuzzi and Hedges 2009; Lin et al. 2017; Wang et al. 2020), there are two important issues: 1) the available cyanobacterial fossils are very rare and 2) cyanobacteria fossils do not provide appropriate maximum calibrations (Wang and Luo 2021). Hence, we also used a recently developed mitochondria-based dating strategy (Wang and Luo 2021) to estimate the origin time of anammox bacteria. This approach, based on mitochondria endosymbiosis, integrates mitochondrial lineages as a sister group to Alphaproteobacteria (Munoz-Gomez et al. 2022), thus taking the advantages of eukaryotic fossils, particularly several maximum calibrations, to improve date estimates. We compiled two gene sets (see supplementary dataset S1.4, Supplementary Material online), one according to the original 24 mitochondria-encoded genes used in (Wang and Luo 2021) (mito24; 6,295 amino acid sites), the other being six (mito6; 1,238 amino acid sites) out of these 24 genes conserved across the bacterial tree of life (see fig. 2*A* and supplementary text section 3.5, Supplementary Material online). These analyses showed that the posterior time of the LCA of anammox bacteria shifted to the present time by 90–500 My (fig. 2), compared with only using the cyanobacteria-fossil-based approach (fig. 2). Despite the large phylogenetic distance between mitochondrial lineages and anammox bacteria, the mitochondria-based dating strategy is still valuable by providing additional time constraints to the origin of anammox bacteria. For example, the “C8 + Euk” scheme effectively constrained the time estimates compared to using a single maximum bound in C8 (fig. 2*B*). Furthermore, the posterior dates obtained by incorporating eukaryotic fossils (“C8 + euk” and “C10 + euk”) displayed less variation across calibration schemes (C8 and C10) based on cyanobacterial fossils (fig. 2*B*). However, we observed less differences in posterior ages between C1 and “C1 + euk ”, as well as between C3 and “C3 + euk” (fig. 2*B*). This is likely due to the use of a maximum constraint to Node2 in C1 and C3 (Node2 calibration: 3.0–2.32 Ga) but not in C8 and C10 (Node2 calibration: >2.32 Ga). In other words, the benefit of including time maxima from eukaryotic lineages might be maximized if no maximum time constraints are applied to bacterial lineages. Strictly speaking no appropriate maxima are available for cyanobacteria although some kinds of maxima were often imposed in previous studies on cyanobacteria evolution. Hence, it is important to note that, in our study, setting a maximum age of the total group cyanobacteria in some dating schemes is just to show the importance of having maximum ages in constraining the posterior ages of anammox bacteria. In general, the broadly consistent time estimates between analyses using only cyanobacterial and using both mitochondrial and cyanobacterial calibrations highlight an origin of anammox bacteria roughly around or shortly after the GOE.

Besides, there remains a possibility that there are unsampled or even extinct lineages of anammox bacteria that diverged earlier than all anammox bacteria analyzed in the present study. This scenario, if true, hints that the first anammox bacterium could have originated before the occurrence of the LCA of sequenced anammox bacteria but later than their split from the sister non-anammox lineage (Node X in fig. 1; up to 2,600 Ma). Actually, a pre-GOE origin of anammox bacteria was also obtained by applying a larger minimum age (3.0 Ga) (Crowe et al. 2013; Cardona 2019; Fournier et al. 2021) to the total group of oxygenic cyanobacteria based on only cyanobacteria fossils (C8–C13 in supplementary fig. S6, Supplementary Material online), or by using the mitochondria-based strategy (fig. 2*B*). Accommodating the above uncertainties, our analyses suggest that the origin of anammox bacteria, and hence the origin of anammox, most likely falls into the 2.7–2.0 Ga interval, or more generally, around the early Proterozoic. Running MCMCTree analysis with no sequence data showed different distributions of time estimates for both the clade of anammox bacteria and the four calibration points (supplementary fig. S7, Supplementary Material online), suggesting that sequence data are informative for our molecular clock analysis. Besides, we estimated similar origin times of anammox at around 2.1 Ga (the AR model) or 2.3 Ga (the IR model; see supplementary fig. S8, Supplementary Material online) with a denser taxon sampling shown in supplementary fig. S2, Supplementary Material online (Genome set 2; see supplementary dataset S1.1, Supplementary Material online). For this analysis, we used a penalized likelihood-based dating algorithm (Sanderson 2002) which is very time-efficient on such large phylogenomic datasets. Note that unlike Bayesian molecular dating software, the likelihood-based method we used only makes point estimations for the node ages (no standard deviation), thereby not allowing for fully appreciating the uncertainties in age estimates and any inference based on that. In general, the above result that two independent methods (Bayesian and likelihood-based) employing distinct numbers of genomes (2,077 vs. 85) estimates a similar origin time of anammox bacteria suggests that taxon sampling may not affect our dating analysis.

### The Geochemical Context of the Origin of Anammox Bacteria

We speculate that the timing of the origin of the anammox metabolism is linked to the increasing availability of nitrite in surface environments, because ammonium was likely present in the deep anoxic ocean throughout the Precambrian (Stueken et al. 2016; Yang et al. 2019) and therefore presumably not a limiting substrate. The first abiotic source of nitrogen oxides on the early Earth, including NO and nitrite, would have been lightning reactions between N_2_ and CO_2_ in the Archean atmosphere (Kasting and Walker 1981; Wong et al. 2017). A prior study suggested that this process led to micromolar levels of nitrite in seawater (Wong et al. 2017), and the supply of NO may have been even higher, considering that it is the initial reaction product of lightning (Kasting and Walker 1981; Wong et al. 2017). This implies the possibility of an earlier origin of anammox bacteria. In fact, an intriguing hypothesis is that NO-dependent anammox (Hu et al. 2019) is ancestral to all anammox bacteria and arose before the appearance of significant nitrite levels with the GOE, perhaps driven by the lightning source of NO. However, the original estimates of the lightning-derived flux of nitrogen oxides may have been based on perhaps unrealistically high amounts of CO_2_ (Charnay et al. 2017). Furthermore, to our knowledge, there has been no isotopic evidence in the early Archean rock record prior to 3 Ga for the presence of a significant nitrite or nitrate reservoir in the early ocean (Stueken et al. 2015; Koehler et al. 2019; Ossa Ossa et al. 2019), and it is conceivable that any nitrogen oxides that were supplied to the ocean by lightning were rapidly reduced to ammonium or N_2_ by ferrous iron, possibly even abiotically (Summers and Chang 1993; Brandes et al. 1998). Hence, although a background lightning flux of nitrogen oxides almost certainly existed, to our knowledge, there seems little evidence that it could supply a reliable metabolic substrate for early life. Furthermore, it is important to note that our phylogenetic results are not supportive of this hypothesis since the NO-utilizing anammox bacteria discovered to date are affiliated with phylogenetically shallow lineages from *Ca.* Kuenenia and *Ca.* Brocadia (fig. 1). To our knowledge, there have been no studies reporting the ability of using NO as the sole electron acceptor in any other lineages of anammox bacteria. Hence, if NO-dependent anammox would have been present in the LCA of anammox bacteria, it was not clear why it was lost in all other anammox bacteria which resulted from multiple independent losses of the trait, a scenario apparently not favored from an evolutionary perspective (as shown by the arrows in supplementary fig. S2, Supplementary Material online). There are also anammox bacteria that are capable of using hydroxylamine as an oxidant; however, this molecule is a highly reactive compound and is usually considered as an intermediate in the nitrogen cycle, thereby rendering it a less important compound from the perspective of geological timescales. We are unaware of any non-biological sources of hydroxylamine. Taken together, our results are inconsistent with an Archean emergence of the anammox metabolism driven by lightning-derived nitrogen oxides. Instead, our results are most parsimoniously explained by the appearance of more significant nitrite/nitrate reservoirs around the time of the GOE.

The first transient appearances of nitrite/nitrate-dependent metabolisms are captured by the sedimentary nitrogen isotope record in late Mesoarchean soils at 3.2 Ga (Homann et al. 2018) and in Neoarchean shallow-marine settings at 2.7 and 2.5 Ga (Garvin et al. 2009; Godfrey and Falkowski 2009; Koehler et al. 2018). These observations may reflect local and/or temporally restricted oxygen oases (Anbar et al. 2007; Lalonde and Konhauser 2015). Widespread nitrite/nitrate availability is inferred for the Paleoproterozoic (2.4–1.8 Ga), that is, in the immediate aftermath of the GOE (Zerkle et al. 2017; Kipp et al. 2018; Luo et al. 2018). Importantly, O_2_ is necessary, although a trace amount is feasible, to extant nitrifying organisms for the oxidation of ammonium to nitrite and nitrate (Lehtovirta-Morley 2018; Cheng et al. 2019; Ranjan et al. 2019). We noticed that a recent study (Kraft et al. 2022) reports the ability of the model ammonia-oxidizing archaeal species (*Nitrosopumilus maritimus*) to continue ammonia oxidation after consuming all supplied O_2_ by NO disproportionation to generate O_2_, but the underlying genetic pathway is not known, making it difficult to extrapolate this mechanism to any other ammonia-oxidizing organisms. Hence the rise of oxygen almost certainly triggered the growth of the nitrite/nitrate reservoir, and therefore potentially provided one of the key substrates for anammox bacteria. We note that also NO would have become more abundant after the GOE, because it is an intermediate product in nitrification and denitrification (Stanton et al. 2018). This would put the possibility of NO-driven anammox back on the table. However, NO levels in seawater are highly variable, and so nitrite would likely have been a more reliable substrate. In any case, it would not violate our conclusion that the appearance of anammox in the Paleoproterozoic was linked to the GOE and directly or indirectly to the rise of marine nitrite. It has been shown that a low concentration of nitrite significantly decreases the rate of N removal by anammox bacteria in reactors (Zhang et al. 2017; Li, Zhuang et al. 2020). In modern OMZ in the Bay of Bengal, which may to some extent serve as analogs for the Paleoproterozoic redox-stratified ocean, the concentration of nitrite, instead of ammonium, was proposed as the rate-limiting factor to the anammox metabolism (Bristow et al. 2017). These imply that the rise of nitrite, which is driven by the rise of O_2_, could have facilitated the appearance of anammox bacteria in the early Proterozoic.

In modern ocean, nitrite concentration is generally very low, but it reaches a primary peak (10–1000 nM) around the base of the photic zone and a secondary peak with even higher levels (at the μM level) at the core of OMZ (Buchwald et al. 2015; Zakem et al. 2018). Although it is commonly mentioned that modern OMZ resembles the primordial ocean (Lyons et al. 2009), we argue that the nitrite levels found in modern OMZ are unlikely representative of the primordial ocean before GOE. Nitrites have different sources in these two peaks: it is mainly derived from aerobic ammonia oxidation carried out primarily by ammonia-oxidizing archaea (AOA) in the photic zone but nitrate respiration in OMZ (Dalsgaard et al. 2012). As noted above, nitrification could have appeared by at least 2.7 Ga, as indicated by nitrogen isotope evidence for local accumulation of nitrate in surface ocean waters (Canfield et al. 2010; Stueken et al. 2016; Zerkle et al. 2017). Accordingly, nitrate, the terminal product of aerobic nitrification, was unlikely sufficiently available before GOE to fuel nitrate respiration and thus cannot drive the accumulation of nitrite.

A recent study inferred that the LCA of AOA dates back to ∼2.3 Ga and first appeared on land, driven by increasing O_2_ concentrations in the atmosphere at that time, and that the expansion of AOA from land to the ocean did not occur until nearly 1.0 Ga (Ren et al. 2019). If true, this hints at a dominant role of ammonia-oxidizing bacteria (AOB) in early ocean, before 2.3 Ga. Consistent with this idea, the vast majority of the early-branching lineages of anammox bacteria (*Ca.* Scalindua) and the sister Planctomycetes lineages of the anammox clade in the phylogenomic tree (fig. 1) are found in marine or groundwater habitats. Likewise, a recent study (Zhao et al. 2022) *in silico* surveyed its occurrence in different environments and reported that the majority of them is derived from groundwater and marine ecosystems. Furthermore, in the 16S rRNA gene tree which represents a greater diversity than the phylogenomic tree, marine lineages still account for the majority of the earliest-branching lineages of anammox bacteria (supplementary fig. S3, Supplementary Material online). Thus, it seems likely that the LCA of anammox bacteria originated in the marine realm where AOB thrived first. The above arguments, although speculative, tentatively suggest that the nitrite demand for anammox had been readily met by GOE by taking advantage of the significant amount of O_2_ newly available, which is broadly consistent with our molecular dating results suggesting an origin of anammox shortly before or after the GOE.

### Genomic Changes Potentially Related to Anammox Metabolism and Habitat Adaptation upon the Origin of Anammox Bacteria

**Fig. 3.**
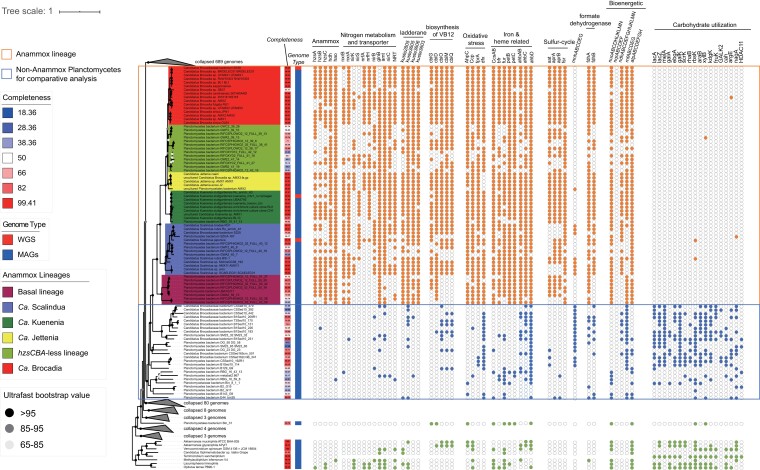
The phyletic pattern of ecologically relevant genes in the comparison between anammox bacteria and non-anammox bacteria. Solid circles at the nodes indicate the ultrafast bootstrap values in 1,000 bootstrapped replicates. Note that copy number difference is not indicated since most genes displayed here are present as a single copy in genome except a few exceptions like *hao* (see supplementary dataset S1.5, Supplementary Material online for the table summarizing the copy number of each gene). The phylogenomic tree on the left was constructed with 887 genomic sequences described in supplementary text section 2.1. Those Planctomycetes genomes not used for comparative genomics analyses are collapsed into grey triangles, and the numbers of collapsed genomes are labeled next to the triangles. The target group and reference group for comparative genomic analysis are within an orange or blue box, separately. For each genome, the genome completeness estimated by CheckM is visualized with a color strip and labeled besides leaf nodes. The right next color strip represents the type of genomic sequences used in our study including MAGs and whole-genome sequencing (WGS) from either enriched culture sample or isolate. The filled and empty circles, respectively, represent the presence and absence of particular genes in corresponding genomes. For gene clusters, only genomes with at least half of the members of the gene cluster are indicated by a filled circle. The classifications of annotated genes are labeled above the gene names. *hzsCBA*, hydrazine synthase subunits C, B, and A; *hdh*, hydrazine dehydrogenase; *hao*, hydroxylamine dehydrogenase; *nxrAB*, nitrite oxidoreductase subunits A and B; *nirK*, copper-containing and NO-forming nitrite reductase; *nirS*, cytochrome NO-forming nitrite reductase; *nrfAH*, ammonia-forming nitrite reductase subunits A and H; *glnB*, nitrogen regulatory protein P-II; *nirC*, nitrite transporter; NRT, nitrate/nitrite transporter; *amt*, ammonium transporter; kuste2805, 3603, 3605–3606, proposed genes relative to the synthetic pathways for ladderane at Rattray et al. (2009); *cbiG*, cobalt-precorrin 5*A* hydrolase; *cbiD*, cobalt-precorrin-5*B*(C1)-methyltransferase; *cbiOMQ*, cobalt/nickel transport system; *AhpC*, peroxiredoxin; *Ccp*, cytochrome *c* peroxidase; *dfx*, superoxide reductase; *fprA*, H_2_*O*-forming enzyme flavoprotein; *CcsAB*, cytochrome c maturation systems; *petB*, Cytochrome b subunit of the bc complex; *petC*, Rieske Fe-S protein; *ahbABCD*, heme biosynthesis; *sat*, sulfate adenylyltransferase; *aprAB*, adenylylsulfate reductase, subunits A and B; *fsr*, sulfite reductase (coenzyme F420); *higB-1*, toxin; *higA-1*, antitoxin; *mnhABCDEG*, multicomponent Na^+^/H^+^ antiporter; *fdhAB*, formate dehydrogenase subunits A and B; *nuo*(A-N), NADH-quinone oxidoreductase; *ndh*(A-N), NAD(*P*)H-quinone oxidoreductase; *nqrABCDEF*, Na^+^-transporting NADH:ubiquinone oxidoreductase; *rnfABCDEG*, Na^+^-translocating ferredoxin:NAD^+^ oxidoreductase; *atp*(A-H), F-type H^+^-transporting ATPase; *lacAZ*, beta-galactosidase; *melA*, galA, alpha-galactosidase; *ebgA*, beta-galactosidase; *galK*, galactokinase;, fructokinase; *fruK*, 1-phosphofructokinase; *rhaB*, rhamnulokinase; rbsK, ribokinase; *araB*, L-ribulokinase; *xylB*, xylulokinase; *kdgK*, 2-dehydro-3-deoxygluconokinase; GALK2, N-acetylgalactosamine kinase; *cah*, cephalosporin-C deacetylase; *argE*, acetylornithine deacetylase; *nagA*; N-acetylglucosamine-6-phosphate deacetylase; *HDAC11*, histone deacetylase 11.

We further explored the genomic changes characterizing the origin of anammox bacteria (see supplementary text section 4, Supplementary Material online). This includes the gains of the aforementioned genes that directly participate in anammox, namely *hzsCBA*, *hdh*, and *hao*. The current view of the anammox metabolism posits that electrons consumed by anammox for carbon fixation are replenished by the oxidation of nitrite to nitrate by nitrite oxidoreductase (NXR) (Kartal and Keltjens 2016), which was inferred to be acquired upon the origin of anammox bacteria (fig. 3). Moreover, multiple auxiliary genes for N assimilatory pathways including N regulatory protein (*glnB*), assimilatory nitrite reductase large subunit (*nirB*), transporters like *amt* (ammonium), *nirC* (nitrite), and NRT family proteins (nitrate/nitrite), were also gained at the origin of anammox bacteria (fig. 3). However, genes for nitrogen-related dissimilatory pathways encoding nitrite reduction to NO (*nirK* or *nirS*) and to ammonium (*nrfAH*), respectively, were likely acquired after the origin of anammox bacteria (fig. 3). Anammox occurs at the membrane of anammoxosome, an organelle predominantly composed of a special type of lipid called ladderane, which helps maintain the proton motive force during the anammox metabolism (Moss et al. 2018). Although the biosynthetic pathway of ladderane is yet to be characterized, a previous study (Rattray et al. 2009) predicted 34 candidate genes responsible for the synthesis of ladderane lipids, four of which were potentially acquired during the origin of anammox bacteria (see supplementary text section 4.2, Supplementary Material online; fig. 3).

Anammox bacteria occur in anoxic habitats and exhibit low oxygen tolerance (Zhang and Okabe 2020). Accordingly, the likely acquired peroxidases include cytochrome c peroxidase (Ccp), which could scavenge hydrogen peroxide in the periplasm (Van Vliet et al. 2002), and the most prevalent peroxidase (Johnson and Hug 2019), thioredoxin-dependent peroxiredoxin (AhpC). Besides, the desulfoferrodoxin (Dfx), which functions as superoxide reductase (SOR) to reduce superoxide to hydrogen peroxide and which is broadly distributed among anaerobic bacteria (Johnson and Hug 2019), was likely acquired during the origin of anammox bacteria (fig. 3). Another acquired gene is *fprA*, which encodes flavo-diiron proteins that scavenge O_2_.

Investigating other metabolisms that were also acquired upon the origin of anammox bacteria allows reconstructing the coeval ecology. Our data show that *sat* (fig. 3), which encodes sulfate adenylyltransferase for assimilatory incorporation of sulfate into bioavailable adenylyl sulfate, as well as *aprAB* and *fsr* (fig. 3) encoding dissimilatory sulfite reductase, were acquired upon the origin of anammox bacteria (fig. 3). Unlike other Planctomycetes, anammox bacteria are generally autotrophs that use the Wood–Ljungdahl pathway for carbon assimilation (Kartal, Keltjens et al. 2011). Consequently, they potentially lost many genes involved in carbohydrate utilization (fig. 3). The metabolic loss is further strengthened by the enrichment analysis where the genes predicted to be lost by the LCA of anammox bacteria are enriched in pathways involving hydrolase activity, intramolecular oxidoreductase activity and carbohydrate kinase activity (fig. 3; see Data availability).

Additionally, iron is a vital element for HZS (Dietl et al. 2015) and HDH (Akram et al. 2019), and *in vitro* studies revealed that increased iron concentrations can promote the growth of anammox bacteria (Bi et al. 2014). The ancient oceans are thought to have been ferruginous (high iron availability) according to sedimentary records of iron speciation across several coeval marine settings from the Archean through most of the Proterozoic (Poulton and Canfield 2011), meaning that large amounts of soluble iron would have been available for anammox bacteria. Interestingly, a series of iron-related genes to make use of the abundant iron from the environment were likely acquired upon the origin of anammox bacteria, such as *fur*, *petBC*, *CCsAB*, and *ahbABCD* (fig. 3). As we know, nearly all known iron acquisition genes were regulated by the gene *fur* (Hantke 2001). Subsequently, the acquired iron could form iron-containing proteins, specifically cytochrome, which are encoded or regulated by the acquired genes *petBC, CCsAB*, and *ahbABCD* (Kartal and Keltjens 2016). Furthermore, we identified that the gene *Bfr* that encodes the oligomeric protein, bacterioferritin involved in the uptake and storage of iron (Keren et al. 2004) was likely acquired during the origin of anammox bacteria (fig. 3), which may help to hoard iron in settings where iron became locally sparse, such as along euxinic (sulfide-rich) or oxic marine margins in the Paleoproterozoic (Poulton et al. 2010). A recent study highlights the vital role of bacterioferritin to the anammox regulation (Peng et al. 2022). It is thought that the appearance of euxinic margins around the time of the GOE trapped iron that upwelled from the deep ocean, leading to the demise of iron oxide deposits (i.e., banded iron formations) (Konhauser et al. 2017). Our results tentatively support this model, if the microenvironments that those primordial anammox bacteria colonized were located near the euxinic–oxic interface where nitrite and ammonium were available, while upwelled iron was titrated out of the water column by freely dissolved hydrogen sulfide.

Taken together, the timelines and corresponding genomic changes of anammox bacteria provide implications for the physiological characteristics of descendant anammox bacteria and the origin of other nitrogen-transforming pathways in the context of an estimated evolutionary timeline of anammox bacteria. For example, genomic data reveal that canonical denitrification genes radiated across the tree of life after the GOE (Parsons et al. 2021), and the gains (supplementary fig. S9, Supplementary Material online) of genes *nrfAH* for dissimilatory nitrate reduction to ammonium at the late-branching lineages (*Ca.* Brocadia and *Ca.* Jettenia) at around 800 Ma (fig. 1) are consistent with the scenario when the nitrite/nitrate availability was increased by the Neoproterozoic Oxygenation Event (Ader et al. 2014). The time estimate and genomic changes of the anammox bacteria have gone some way towards enhancing the understanding of the physiological characteristics of modern anammox bacteria and the historical N cycle.

We further explored the sources of the genes acquired by anammox bacteria discussed above. The majority of them formed a monophyletic lineage (supplementary fig. S10, Supplementary Material online). We classified the evolutionary sources of these genes into the following three types: horizontal gene transfer (HGT), ancient duplication, and *de novo* birth. A significant proportion of genes were clustered with other Planctomycetes. These genes might be vertically descended from ancestral planctomycetes or horizontally transferred from other Planctomycetes lineages to anammox bacteria which, we temporarily classified as within-Planctomycetes HGT. Moreover, a majority of the remaining genes seemed to be gained by HGT from a different phylum (supplementary text section 4.4; supplementary fig. S10, Supplementary Material online). Particularly, Firmicutes, Proteobacteria, and Euryarchaeota are the three most important donor lineages of these horizontally transferred genes (supplementary fig. S10, Supplementary Material online). This highlights the role of HGT in enhancing nitrogen assimilation and helping establish the anammox pathway in the origin of anammox bacteria. Note also that the inference could be affected by the limited resolution of gene phylogenies, for example, gene *cbiD* from anammox bacteria is clustered with a small group of Firmicutes, but most Firmicutes branched somewhere else. Thus, caution needs to be taken when interpreting the result.

## Caveats and Concluding Remarks

In the present study, we link an estimated evolutionary timeline of anammox and geological context of early Earth on a simplified view that nitrite-dependent anammox is ancestral to anammox bacteria. However, there are several limitations. First, dating the bacterial evolution has many challenges, which, among others, include the paucity of appropriate fossils, and a long evolutionary distance between anammox bacteria and cyanobacteria (Wang and Luo 2021). In addition to the traditional cyanobacteria-based approach, our study takes the advantages of eukaryotic fossils by implementing a recently developed mitochondria-based approach to provide more robust time estimates (Wang and Luo 2021). Nevertheless, due to the large evolutionary distance between mitochondrial lineages and Planctomycetes, we applied only six mitochondria-encoded orthologs shared by both mitochondrial lineages and Planctomycetes. Moreover, it is worth pointing out the metabolism called feammox, a process where ferric instead of nitrite is taken as the electron acceptor for ammonia oxidation. Both feammox and anammox can anaerobically convert ammonia into dinitrogen (Li et al. 2018), but the former process is supposed to be carried out by Acidimicrobiaceae from the Actinobacteria phylum (Wan et al. 2022; Zhu et al. 2021) and under anaerobic environments (Yang et al. 2021). Likewise, a recent experimental study (Shaw et al. 2020) proposed extant anammox bacteria as electroactive organisms and suggested the feasibility to utilize graphene oxides and man-made metallic electrodes mimicking metal oxides as the electron acceptor. However, the lack of detailed molecular mechanism hampers the inference of its evolutionary history. In any case, metal oxides such as Fe_2_O_3_ or MnO_2_ also became more abundant in the Neoarchean to Paleoproterozoic, that is, around the time of the GOE (Johnson et al. 2016; Konhauser et al. 2017), and hence even if these oxides were used as substrates for anammox, it would not violate our overall conclusion that the GOE played a primary role in driving the evolution of this metabolic pathway. However, it is also worth noting that metal oxides are solids and thus require extracellular electron transfer (Shaw et al. 2020). An entirely intracellular metabolism, using dissolved nitrite, may be more likely to evolve first, especially given that oxygen became bioavailable in the ocean around the same time (see above).

Another important aspect to highlight about our approach is that, our analysis began with inferring the LCA of anammox bacteria based on the presence of *hzsCBA*, a common strategy in phylogenetic studies. It is important to note that such an enzyme complex encoded by multiple genes might originate in different genomic backgrounds and were presented together in a suitable genomic background later in evolution. This scenario, if true, indicates that the appearance of any single subunit of *hzsCBA* genes, which could not perform hydrazine synthesis, should pre-date the LCA of anammox bacteria. Nevertheless, the alternative hypothesis would not affect our inference and following analysis since we only focus on the inferred ancestor of extant anammox bacteria, a strategy commonly used in modern phylogenetic analysis. These alternative possibilities, although not affecting the molecular clock analysis, should be carefully considered when making inference based on our estimated evolutionary timeline of anammox bacteria.

Here, using molecular dating and comparative genomics approaches, we link the emergence of an obligate anaerobic bacterial group, which drives the loss of fixed N in many environments, to the rise of O_2_, and highlight their evolutionary responses to major environmental disturbances. Apparently, the GOE opened novel niches for the origin and subsequent expansion of diverse aerobic prokaryotes such as AOA and AOB, which in turn facilitated the origin of other bacterial lineages, including some anaerobes like the anammox bacteria, by providing the resources for their energy conservation. Our results open up the possibility that a significant proportion of the Proterozoic nitrite budget was consumed by anammox bacteria, and that the sedimentary nitrogen isotope record might be influenced by their activity. Our findings may have implications for Proterozoic climate, because N_2_O has previously been invoked as an important greenhouse gas at that time (Buick 2007), but a relatively higher contribution of anammox bacteria to the marine nitrogen cycle may have hampered N_2_O production (Li, Ling et al. 2020). Finally, our study suggests that the impacts of the GOE go well beyond aerobic prokaryotes. We therefore conclude that molecular dating is a likely feasible approach to complement isotopic evidence for resolving the timeline of biological evolution and for providing additional constraints on climate models of the distant past.

## Materials and Methods

Overall, we compiled two genome sets and one 16S rRNA gene set in our study (supplementary dataset S1.1, Supplementary Material online). A phylogenomic tree (supplementary fig. S1, Supplementary Material online) was generated based on the concatenated alignment of 120 ubiquitous proteins (bac120; supplementary dataset S1.3, Supplementary Material online) proposed for tree inference by the Genome Taxonomy Database (Parks et al. 2017). All published genomic sequences (952 in total) affiliated with the phylum Planctomycetes in NCBI Genbank up to December 2019 were retrieved (supplementary dataset S1.2, Supplementary Material online). To further examine whether the patterns obtained with this Planctomycetes dataset hold the same, we further constructed an expanded set of Planctomycetes genomes by retrieving a total of 2,077 Planctomycetes genomes released by April 2021 at Genbank (supplementary fig. S2, Supplementary Material online; see Data availability). Key anammox genes *hzsCBA* and *hdh* were identified against manually curated reference sequences by BLASTP. For each gene, identified protein sequences were aligned using MAFFT (v7.222) (Katoh and Standley 2013) and the alignments were refined by trimAl (v1.4) (Capella-Gutierrez et al. 2009). All phylogenies were constructed by IQ-tree (v1.6.2) (Nguyen et al. 2015) with substitution models automatically selected by ModelFinder (Kalyaanamoorthy et al. 2017) and branch support assessed with 1,000 ultrafast bootstrap replicates. Note that the constraint topologies for dating analysis (fig. 1; supplementary fig. S5, Supplementary Material online) were inferred with the profile mixture model (LG + C60 + G) which better accommodates across-site heterogeneity in deep-time evolution. Following manual curation, four well-recognized genera and two separate lineages of anammox bacteria were highlighted with different colors, and the presence of key genes were annotated with filled symbols beside the labels (supplementary fig. S1, Supplementary Material online). Furthermore, the phylogenomic tree was generated with similar methods using 16S rRNA genes identified from downloaded genomes and those retrieved from the SILVA database (supplementary fig. S3; supplementary dataset S1.3, Supplementary Material online). All trees (including phylogenomic and 16S trees) in our study were visualized with iTOL (Letunic and Bork 2019). The specific parameters of the above analysis are shown in supplementary text section 2, Supplementary Material online.

Molecular dating analysis was carried out using the program MCMCTree from the PAML package (4.9j) (Yang 2007). In our study, 13 calibration sets constructed with different time constraints of the root and three calibration nodes within the cyanobacteria lineage were used (see supplemental text section 3.2, Supplementary Material online). The topology constraint for dating analysis was generated using 85 genomes, which were sampled from the phylogenomic tree of phylum Planctomycetes (see supplemental text section 3.1, Supplementary Material online), and the model LG + C20 + F + G under posterior mean site frequency (PMSF) approximation (Wang et al. 2018). To perform dating analysis, clock models and different calibration sets were compared in 26 schemes (see supplemental text section 3; supplementary dataset S2.1, Supplementary Material online) (Dos Reis et al. 2016; Reis et al. 2018). For each scheme, the approximate likelihood method (Dos Reis and Yang 2011) of MCMCTree were conducted in duplicate with identical iteration parameters (burn-in: 10,000; sample frequency: 20; number of sample: 20,000). The convergence of each scheme was evaluated by comparing the posterior dates of two independent runs (see Data availability). With the updated genome set 2 (supplementary dataset S1.1, Supplementary Material online), we repeated the taxon sampling process and generated an alternative phylogeny for dating analysis (supplementary fig. S5, Supplementary Material online) using LG + C20 + F + G model (C20: 20 classes of site-specific amino acid profiles) under PMSF approximations. Similarly, we also generated another phylogeny for dating analysis using the same genome set as used in Figure 1 but with the profile mixture model (LG + C60 + G) which uses 60 classes of amino acid profiles (Le et al. 2008). Note that the missing parameter + F would not significantly change the results since it only adds another profile amino acid calculated from the original data. These two dating analyses were conducted with the dating scheme C1 (IR model). Moreover, we performed likelihood-based dating analyses (Sanderson 2002) (supplementary fig. S8, Supplementary Material online) using the phylogenomic tree (supplementary fig. S2, Supplementary Material online) and the calibration C1 (see Data availability) implemented in ape (Paradis and Schliep 2019).

The phylogenetic differences between gene trees and the species tree were reconciliated by GeneRax (Morel et al. 2020) with unrooted gene tree as input and automatically optimized duplication, transfer, loss (DTL) rates. For each gene identified from the genome set 2 (supplementary dataset S1.1, Supplementary Material online), we used a species tree comprised by all anammox bacteria pruned from the phylogenomic tree (supplementary fig. S2, Supplementary Material online) as the reference. We used recommended parameters including SPR strategy, undated DTL model and a maximum radius of five.

For comparative genomics analysis, the protein-coding sequences were annotated against KEGG (Kanehisa and Goto 2000), CDD (Lu et al. 2020), InterPro (Mitchell et al. 2019), Pfam (El-Gebali et al. 2019), TIGRFAM (Haft et al. 2001) and TCDB (Saier et al. 2016), individually (see supplemental text section 4, Supplementary Material online). Following annotations, the potentially gained/lost genes between groups (anammox bacteria vs. non-anammox bacteria; an anammox bacterial genus vs. all other anammox bacteria) were statistically tested using two-sided fisher's exact tests. Finally, the resulting *P*-values were corrected with the Benjamini–Hochberg FDR (false discovery rate) procedure. Specifically, genes with corrected *P*-values smaller than 0.05 and with larger ratio in the study group were defined as gained genes, and genes with corrected *P-*values smaller than 0.05 and with smaller ratio in the study group were defined as potentially lost genes. Those potentially gained/lost genes were summarized for their detailed functions (see Data availability).

## Supplementary Material

msac170_Supplementary_Data

## Data Availability

All used genomic sequences, generated phylogenetic trees, estimated divergence times and the used python codes are deposited in the online repository https://github.com/luolab-cuhk/anammox-origin.
